# Temperature regime during embryogenesis alters subsequent behavioural phenotypes of juvenile brown trout

**DOI:** 10.1098/rsbl.2022.0369

**Published:** 2022-11-30

**Authors:** Kunio Takatsu, Oliver M. Selz, Jakob Brodersen

**Affiliations:** ^1^ Department of Fish Ecology and Evolution, Center for Ecology, Evolution & Biogeochemistry, Eawag: Swiss Federal Institute of Aquatic Science and Technology, 6047 Kastanienbaum, Switzerland; ^2^ Federal Office for the Environment (FOEN), Aquatic Restoration and Fisheries Section, 3011 Bern, Switzerland; ^3^ Department of Aquatic Ecology & Evolution, Institute of Ecology and Evolution, University of Bern, 3012 Bern, Switzerland

**Keywords:** behaviour, embryonic development, hydropower-induced temperature changes, climate change, *Salmo trutta*, thermal plasticity

## Abstract

Climate warming imposes a serious threat, especially to freshwater ecosystems in temperate and (sub)polar regions, which are often dominated by cold-adapted ectotherms. Although relatively intense warming during winter is common across the climatic regions, comparably little focus has been put on the organismal impacts of winter warming. Embryonic development, which is exceptionally susceptible to ambient temperature, occurs during winter in various freshwater ectotherms. Yet, our knowledge of the effects of increased temperature during embryogenesis on later life stages is limited. Using brown trout (*Salmo trutta*), we examined how a 1.5°C temperature increase from fertilization to hatching affects various traits at the onset of the free-swimming stage (i.e. a comparison between 3.5 and 5.0°C treatments). Although all hatchlings were kept at the same temperature (7.0°C) from hatching to the onset of the free-swimming stage for about two months, the temperature increase during embryogenesis substantially reduced key ecological behaviours, i.e. activity and exploration levels, at the onset of the free-swimming stage despite only marginal temperature effects on morphological and physiological traits at this stage. Given the importance of behavioural traits in early growth and survival, our study suggests a likely pathway through which subtle changes in mean winter temperature affect early fitness.

## 1. Introduction

Ambient temperature is an important abiotic factor influencing ectotherms' behavioural traits, such as activity and exploration levels [1], which affect prey capture and predator avoidance, and in turn, growth and survival [2–4]. Acceleration of metabolic processes due to increased temperature is common among ectotherms, resulting in a change in activity and explorative patterns [1,5,6]. Besides the immediate effect on physiology, the temperatures to which individuals have been exposed during their development affect various traits, such as body size, organ size and morphology, which again result in a change in activity and exploration levels [1,7,8]. Despite this interaction, more is known about the immediate direct effect of temperature on behavioural traits than how different temperatures during development affect life-history traits and, subsequently, behavioural traits later in life [1].

Notably, our knowledge of how temperatures during development affect behavioural traits exhibited later in life is mostly limited to that obtained from studies in which mean rearing temperatures are kept constant and non-variable across all life stages (i.e. from fertilization to sexual maturation; [7–9]). Such an approach is a useful first step in investigating the effects of temperatures during development on behavioural traits. However, on the other hand, it might be an oversimplified approach considering the natural and anthropogenically altered temporal variation in temperatures [10–12]. This is especially true for ectotherms in temperate and (sub)polar regions, where ontogenetic development is often closely associated with seasonality. This considered, investigating how temperatures during short time windows of development affect subsequent behavioural traits is an essential next step. To address this question, salmonids, representative fishes of temperate and (sub)polar regions, are excellent model organisms.

Relatively intense warming during winter is common across temperate and (sub)polar regions [10], and the trend is further exaggerated, especially in alpine streams inhabited by salmonid fishes, by anthropogenic activities, e.g. hydropower plants [12,13]. Importantly, embryonic development, the most environmentally susceptible period [14,15], occurs during winter in most salmonid fishes [16–18]. Given that an increase in temperature during embryonic development affects not only timing and body size at hatching but also muscle and organ development [19–22], it could also eventually affect behavioural traits exhibited later in life. Here, we report results from a laboratory experiment using brown trout (*Salmo trutta*) embryos specifically aimed at examining how temperatures during embryogenesis (i.e. from fertilization to hatching) affect the behavioural traits at the onset of the free-swimming stage—key components determining early survival and growth of salmonid fishes [4,23,24].

## 2. Methods

For detailed descriptions of the methods, see the electronic supplementary material, S1 and S2.

### (a) Experimental design

**Figure 1.**
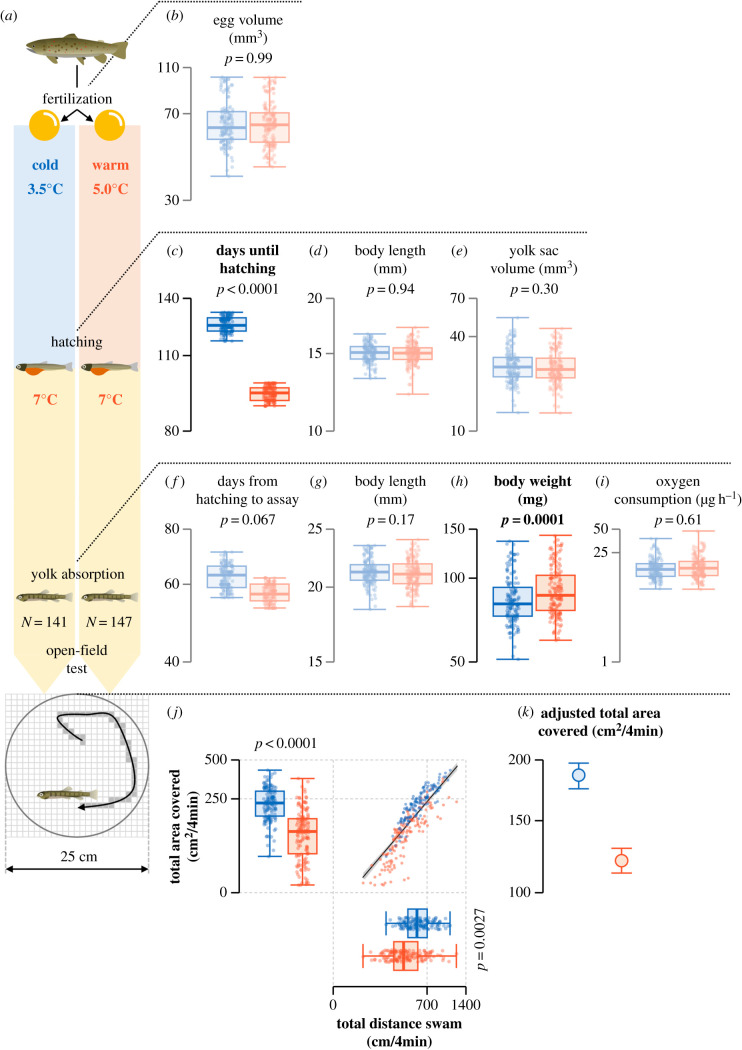
(*a*) Schematic diagram of the experiment. (*b–k*) Overview of early life-history traits and behavioural traits in the cold (shown in blue) and warm (shown in red) treatments. In (*b–j*), dots represent individuals, the thick horizontal bars represent the median, the box contains 50% of the data and the whiskers indicate the range. The solid line in (*j*) is the predicted relationship between total distance swum and total area covered obtained from the linear mixed model, and the shaded area is the 95% CI. In (*k*), the filled circles denote mean total area covered adjusted for mean total distance swum, and error bars denote 95% CI. Note that *y*-axes are log-scaled in (*b–i*) and square-root scaled in (*j–k*). Traits for which a significant effect of temperature treatment was detected are depicted in bright colours in the figures and labelled in bold.

Each of 64 eggs collected from each of 20 full- or half-sib families consisting of two males and 20 females (i.e. 1280 eggs in total) of broodstock brown trout at Flüelen hatchery in the Swiss canton of Uri on 7 December 2019 was kept separately until yolk absorption using eight vertical incubators each consisting of eight trays. Each of the 64 trays contained an egg from each of the 20 families. Of the eight incubators, four were set at 3.5°C (i.e. cold treatment) and the remaining four at 5°C (i.e. warm treatment) (figure 1*a*). The 1.5°C difference in temperature during embryonic development is equivalent to or smaller than the temperature changes caused by climate change and anthropogenic activities (e.g. deforestation, urbanization and hydropower plants [12,13,25,26]).

After half of the eggs hatched in each incubator, we increased the temperature by 1°C every day until it reached 7°C. After that, hatchlings were kept at 7°C without food supplementation for the entire duration from hatching to yolk absorption for the subsequent behavioural assay (figure 1*a*). All chosen temperatures in this study, including the behavioural assay, were within the suitable temperature range for early development [16]. Indeed, the survival rate of embryos was high during the experiment and did not significantly differ between temperature treatments (see electronic supplementary material, S1). Specifically, hatching rates in the warm and cold treatments were 73 and 70%, respectively, and survival rates from hatching to the behavioural assay were 90 and 88%, respectively. During the rearing experiment, we measured the following eight traits: (1) the volume (mm^3^) of eggs just before assigning them to the incubators, (2) the number of days from fertilization to hatching, (3) body length (mm) and (4) yolk sac volume (mm^3^) at hatching, (5) routine metabolic rate within two weeks before the start of the behavioural assay, (6) days from hatching to behavioural assay, and (7) body length (mm) and (8) body weight (mg) after the third trial of the behavioural assay (see below and figure 1*b–i*). For the routine metabolic rate (i.e. the metabolic rate of an undisturbed and spontaneously active individual [27]), we measured the oxygen consumption rate (O_2_ µg h^−1^) of hatchlings at 7°C using respirometry chambers.

### (b) Open-field behavioural assay

For the behavioural assay, hatchlings originating from the largest and smallest eggs of each of the 20 families in each of the eight incubators were used to cover the size range of hatchlings in each family (except when there was only one surviving hatchling of a family in an incubator). As a result, we assayed behavioural traits of 147 and 141 hatchlings from the warm and cold treatments, respectively. For each temperature treatment, we performed a behavioural assay when all selected hatchlings absorbed their yolk sac almost completely (i.e. the onset of the free-swimming and exogenous feeding stage) (figure 1*a*). As in the rearing experiment, we did not feed hatchlings during the behavioural assay.

The behavioural assay was performed in a temperature-controlled room, where the water temperature was maintained at 9.1 ± 0.1°C (*N* = 6). The undisturbed swimming patterns of hatchlings were videotaped for 5.5 min in an open-field (25 cm in diameter bucket filled with 2 l of oxygen-saturated water (4.5 cm in depth) (figure 1*a*)) once a day for three consecutive days (i.e. 5–7 May 2020 in the warm treatment and 11–13 June 2020 in the cold treatment). By analysing the videos, total distance swum (cm) and total area covered (cm^2^) during 4 min were measured (i.e. we did not use the first 60 and last 30 s in the videos for analysis) (figure 1*a*). Water was exchanged in each bucket after each assay.

### (c) Statistical analyses

Since focal behavioural traits were highly repeatable (see electronic supplementary material, S2), we used individual-mean values of the traits for the following statistical analyses.

#### (i) Effects of temperature during embryogenesis on swimming patterns

We performed linear mixed models (LMMs) using the lmer function in the lme4 package [28] to assess how temperature treatment affects focal behavioural traits. Prior to the analyses, individual-mean values of focal behavioural traits were square-root transformed to meet the assumptions of linear models. In all models, we considered the number of days from hatching to behavioural assay (log-transformed) as a covariate to account for behavioural differences caused by developmental stages. Furthermore, for the total area covered, we considered total distance swum as an additional covariate. This allowed us to examine whether there were differences in swimming trajectories and, thus, exploration levels between temperature treatments. For example, for a given total distance swum, a higher total area covered can be interpreted as a higher exploration level. We considered female ID and tray level nested within incubator ID as random factors to account for non-independence among hatchlings sharing the same mother and rearing water, respectively. In all LMMs in this study, when the variance of a random factor was close to zero and produced models with a singular fit, we removed the random factor from the models.

#### (ii) Exploration of mechanisms underlying the temperature effects

**Figure 2.**
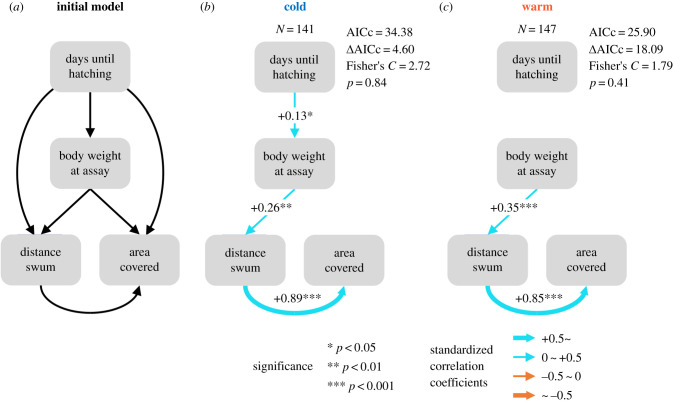
(*a*) A schematic representation of our hypothesized causal relationship between days from fertilization to hatching, body weight (mg) at behavioural assay, and behavioural traits (distance, cm; area, cm^2^). Final path models in (*b*) cold and (*c*) warm treatments. AICc, corrected Akaike information criterion.

First, to explore key early life-history traits in differentiating behavioural traits between temperature treatments, we performed LMMs on the following traits: (1) egg volume, (2–4) traits at hatching (number of days from fertilization to hatching, body length and yolk sac volume), (5–8) traits at behavioural assay (number of days from hatching to behavioural assay, body length, body weight and oxygen consumption rate) (figure 1*b–i*). All trait values were log-transformed. Female ID was considered as a random factor. In addition, except for egg volume, tray level nested within incubator ID was also considered as a random factor. Then, by using the early life-history traits in which a significant difference between temperature treatments was detected and behavioural traits, we constructed piecewise structural equation models (piecewiseSEM [29]) for each treatment to explore how early life-history traits affect behavioural traits (figure 2). For detailed descriptions of the statistical analyses, see electronic supplementary material, S3.

## 3. Results

### (a) Effects of temperature during embryogenesis on swimming patterns

Total distance swum in the warm treatment (439.8 ± 17.2 cm (mean ± s.e.)) was 25% lower than in the cold treatment (589.2 ± 15.3 cm) (*F*_1,6.7_ = 21.3, *p* = 0.003) (figure 1*j*). Similarly, total area covered in the warm treatment (112.7 ± 6.6 cm^2^) was 51% lower than in the cold treatment (229.6 ± 6.7 cm^2^) (figure 1*j*). Importantly, total area covered was significantly lower in the warm treatment than in the cold treatment (*F*_1,92.5_ = 67.0, *p* < 0.0001) even after accounting for total distance swum (*F*_1,283.8_ = 828.2, *p* < 0.0001) (figure 1*j,k*). The number of days from hatching to behavioural assay had no significant effect on total distance swum (*F*_1,13.5_ = 3.3, *p* = 0.09) and total area covered (*F*_1,96.8_ = 0.2, *p* = 0.7).

### (b) Exploration of the mechanisms underlying the temperature effects

Among the eight measured early life-history traits (figure 1*b–i*), we found significant differences between temperature treatments only in the number of days from fertilization to hatching and in body weight at behavioural assay. The number of days until hatching and the body weight in the warm treatment were 25% lower (*F*_1,6.0_ = 184.2, *p* < 0.0001) and 8% higher (*F*_1,46.0_ = 26.6, *p* < 0.0001) than in the cold treatment, respectively.

In the piecewiseSEM for each temperature treatment, we initially considered all possible links between early life-history traits in which we detected significant effects of temperature treatment (i.e. number of days until hatching and body weight at behavioural assay (see above and figure 1*c,h*)) and behavioural traits (figure 2*a*). Subsequent backward model selection revealed that body weight at behavioural assay was a key early life-history trait explaining variation in behavioural traits within each temperature treatment (figure 2*b,c*). Specifically, in both temperature treatments, heavier hatchlings swam longer distances than lighter hatchlings, and hatchlings that swam longer distances covered larger areas than those that swam shorter distances. Hence, an increase in body weight indirectly increased total area covered in each temperature treatment. However, despite the higher body weight of hatchlings in the warm treatment compared with the cold treatment (figure 1*h*), both activity and exploration levels in the warm treatment were lower than in the cold treatment (figure 1*j*). Thus, the temperature effects on body weight do not seem to explain the lower activity and exploration levels found in the warm treatment.

## 4. Discussion

In the present study using brown trout as a model organism, we have demonstrated that an increase in temperature only during embryonic development results in individuals being markedly less active and explorative at the onset of the free-swimming stage. This suggests that temperature conditions during embryogenesis are critical in shaping early behavioural traits, key components determining individual early survival and growth [3,4,23,24]. In many salmonid fishes, embryonic development occurs during winter [16–18], when relatively intense warming occurs as a result of climate change and anthropogenic activities [10,12,13]. Our study suggests a likely pathway through which such an increase in winter temperature affects early growth and survival of the ecologically and commercially important fish in temperate and (sub)polar streams. As represented by salmonid fishes, ontogenetic development of ectotherms in temperate and (sub)polar regions is often closely associated with seasonality. All this considered, the present study emphasizes the importance of gaining detailed knowledge of temperature effects on different life stages in order to better assess the impacts of climate change and anthropogenic activities on ectotherm populations in the climatic regions.

In the present study, we were not able to elucidate the underlying mechanisms differentiating behavioural traits between temperature treatments. Body size and metabolic rate, which are generally susceptible to temperature during development, have been thought to be key traits in differentiating behavioural traits of individuals kept in contrasting temperatures during development [1]. However, we could not detect any effect of temperature treatment on metabolic rate. Furthermore, although we found that (i) the body weight of hatchlings from the warm treatment was higher than those from the cold treatment and (ii) an increase in body weight increased activity and exploration levels in each temperature treatment, hatchlings from the warm treatment were less active and explorative than those from the cold treatment. Therefore, both metabolic rate and body weight were unlikely to explain the observed behavioural differences between temperature treatments. The discrepancy in key traits in differentiating behavioural traits between studies emphasizes the complicated processes that shape behavioural traits. Although not measured in the present study, there are several possible traits that could influence behavioural traits and explain behavioural differences between temperature treatments. In salmonid fishes, muscle, organ and brain development, which could influence behavioural traits, are also susceptible to rearing environment during embryogenesis, including temperature [21,22,30,31]. Moreover, the use of yolk sac resources could differ depending on rearing temperatures during embryonic development [32,33], possibly influencing the nutritional status of hatchlings. Given that nutritional status is a key component determining individual behaviour [34–36], the temperature-dependent use of yolk sac resources could also eventually influence the behavioural traits of hatchlings. Anatomical, histological and chemical investigations on hatchlings kept in contrasting temperatures during embryogenesis may provide insight into the mechanisms differentiating behavioural traits between temperature treatments.

Becoming less active and explorative in response to warmer temperatures during embryogenesis could be interpreted as adaptive behavioural plasticity. In salmonid fishes, the growth period for hatchlings is generally limited to the spring to autumn period. Importantly, smaller individuals at the onset of winter often have lower energy reserves relative to their metabolic rate, which often results in higher winter mortality rates than for larger individuals in various species, including salmonid fishes [37–39]. As shown in this and previous studies [18–20], warmer temperatures during embryogenesis can shorten the development period from fertilization to hatching, extending the duration of the growth period but simultaneously increasing the time exposed to predation risk. Although the relationships between the behavioural traits and growth and survival of individuals are mixed and controversial [2–4,6,40,41], it has been generally assumed that less active and explorative individuals are less exposed to predation risk while they are less efficient in food intake and, eventually grow slowly [42,43]. All this considered, if the extension of the growth period allows hatchlings to grow large enough to overwinter regardless of activity and explorative levels, the reduction in activity and exploration levels in response to warmer temperatures during embryogenesis could be favoured since the predation risk would be reduced. At the same time, however, warmer temperatures during embryogenesis, which occurs in winter, may also affect the densities and traits of predators and prey of the hatchlings [44]. This will further complicate the effects of the reduced activity and exploration levels on early growth and survival. Examining (i) the extent to which the reduced activity and exploration levels caused by an increase in winter temperature persist, (ii) how the behavioural changes affect early growth and survival during the extended growth period and time exposed to predation, and (iii) how the effects of the behavioural changes on early fitness differ depending on community members are important next steps in understanding the organismal impacts of winter warming on ecologically and commercially important fish species.

## Data Availability

Data are available from the Dryad Digital Repository: https://doi.org/10.5061/dryad.fttdz08vz [45]. Supplementary material is available online [46].
